# Divergent developmental trajectories in two siblings with neuropathic mucopolysaccharidosis type II (Hunter syndrome) receiving conventional and novel enzyme replacement therapies: A case report

**DOI:** 10.1002/jmd2.12239

**Published:** 2021-07-27

**Authors:** Kazuyoshi Tomita, Shungo Okamoto, Toshiyuki Seto, Takashi Hamazaki, Sairei So, Tatsuyoshi Yamamoto, Kazunori Tanizawa, Hiroyuki Sonoda, Yuji Sato

**Affiliations:** ^1^ Department of Pediatrics Osaka City University Graduate School of Medicine Japan; ^2^ JCR Pharmaceuticals Co., Ltd. Japan

**Keywords:** enzyme replacement therapy, Hunter syndrome, idursulfase, mucopolysaccharidosis II, neurocognitive development, pabinafusp alfa

## Abstract

Mucopolysaccharidosis type II (MPS II; Hunter syndrome) is an X‐linked recessive lysosomal storage disease caused by a mutation in the *IDS* gene and characterized by systemic accumulations of glycosaminoglycans. Its somatic symptoms can be relieved by enzyme replacement therapy (ERT) with idursulfase, but because the enzyme cannot cross the blood‐brain‐barrier (BBB), it does not address the progressive neurodegeneration and subsequent central nervous system (CNS) manifestations seen in patients with neuropathic MPS‐II. However, pabinafusp alfa, a human iduronate‐2‐sulfatase (IDS) fused with a BBB‐crossing anti‐transferrin receptor antibody, has been shown to be efficacious against both the somatic and CNS symptoms of MPS II. We report two cases of MPS‐II in Japanese siblings sharing the same G140V mutation in the *IDS* gene, who showed markedly contrasting developmental trajectories following enzyme replacement therapy (ERT). Sibling 1 was diagnosed at 2 years of age, started undergoing conventional ERT shortly afterward, and scored a developmental quotient (DQ) of 53 on the Kyoto Scale of Psychological Development (KSPD) at 4 years of age. Sibling 2 was diagnosed prenatally and received conventional ERT from the age of 1 month through 1 year and 11 months, when he switched to pabinafusp alpha. He attained a DQ of 104 at age 3 years and 11 months, along with significant declines in heparan sulfate concentrations in the cerebrospinal fluid. This marked difference in neurocognitive development highlights the importance of early initiation of ERT with a BBB‐penetrating enzyme in patients with neuropathic MPS‐II.

**Synopsis**

Early initiation of enzyme replacement therapy with a blood‐brain‐barrier penetrating enzyme can induce improvements in the neurodevelopmental trajectory of patients with neuropathic mucopolysaccharidosis type II.

## 1 INTRODUCTION

Mucopolysaccharidosis II (MPS‐II; Hunter syndrome) is an X‐linked recessive lysosomal storage disorder caused by mutations in the *IDS* gene which leads to a deficiency in iduronate‐2‐sulfatase (IDS),^1^ an essential enzyme for the catabolism of glycosaminoglycans (GAGs), such as heparan sulfate (HS) and dermatan sulfate. Systemic pathological accumulations of GAGs in the lysosomes of patients with MPS‐II lead to multiple peripheral/somatic symptoms

^2^, ^3^ and complex progressive neurodegeneration in the central nervous system (CNS).^4^ The clinical presentation of MPS‐II is highly variable with a wide spectrum of severity,^5^ but it is broadly categorized into the severe (neuropathic) phenotype which affects around two‐third of the patient population.^2^, ^3^ In severe type patients, growth retardation shown at early infancy such as speech and walking difficulty observed from the age of 10 years onward, leading to significant activity decline rendering requiring total assistance, **bedridden** need of, and respiratory assistance.^2^, ^3^, ^5^ Sever type patients who remain untreated, cardiovascular and respiratory symptoms result in death by the age of 20 years.^6^, ^7^ On the other hand, attenuated (non‐neuropathic) phenotype patients, which involves few or no CNS manifestations, show only a small number of symptoms, such as joint contracture, and follow varied disease courses, resulting in some cases not being diagnosed until adulthood. Previous studies have suggested that patients with deletions, recombination, frameshift, and splicing mutations have a severe phenotype, with many exceptions and opacity.^8^ The incidence of MPS II is estimated to be 0.13 to 1.07 per 100  000 births.^9^

ERT with recombinant human IDS (idursulfase and idursulfase beta) has been available for patients with MPS‐II since 2006. It is effective against the somatic symptoms, but because intravenously administrated idursulfase cannot cross the blood‐brain barrier (BBB) to address the neurodegeneration seen in patients with the severe subtype, it is unable to improve the CNS manifestations in these patients, who often do not live beyond their second decade.^6^, ^7^ Much research has been done, therefore, with the aim of devising treatment for these hitherto intractable CNS manifestations.

Pabinafusp alfa (JR‐141), a BBB‐penetrating fusion protein that consists of intact human IDS and an anti‐human transferrin receptor antibody, reaches the brain parenchyma by transcytosis via transferrin receptors on the cerebrovascular endothelial cells,^10^ and it has been shown in clinical trials in Japan and Brazil to be efficacious against both somatic and CNS symptoms.^11^, ^12^, ^13^ We describe herein two cases of MPS‐II in Japanese siblings sharing the same G140V mutation in the *IDS* gene. Both underwent intravenous ERT with idursulfase, but the younger sibling later switched to pabinafusp alfa and showed a markedly improved neurodevelopmental trajectory over that of his older brother, suggesting that early initiation of ERT with a BBB‐crossing enzyme plays a critical role in treating patients with the severe subtype of MPS‐II.

## 2 CASE PRESENTATION

We report two cases of MPS‐II in male Japanese siblings aged 9 and 4 years at the time of writing who both have the same G140V mutation in the *IDS* gene. The older, sibling 1, was born at term (40 months 1 week) with a birth weight of 3860 g following an uneventful pregnancy. Left hydronephrosis had been detected prenatally on echography, and right hydrotestis and a left inguinal hernia 1 month postnatally. Bilateral laparoscopic herniorrhaphy was successfully performed when the patient was 2 months old, and there has been no recurrence. MPS‐II was diagnosed when he was 2 years and 4 months old; conventional weekly ERT with intravenous administration of idursulfase was started and is still being given.

The younger boy, sibling 2, was born by normal delivery after an uncomplicated pregnancy of 40 months and 1 week, weighing 3490 g. He had been prenatally diagnosed with MPS‐II, and at 5 days old, his plasma IDS activity was below the detection limit. Physical examination at 1 month revealed a left inguinal hernia and slight hepatomegaly, along with an atrial septal defect detected on echocardiography. He received conventional weekly intravenous ERT with idursulfase from 1 month after birth until age 1 year and 11 months, when he was enrolled in a phase 2/3 clinical trial of pabinafusp alfa,^12^ during which he received intravenous administration of the trial drug at a weekly dose of 2 mg/kg for about 2 years. Bilateral inguinal herniorrhaphy was performed when he was 3 months old, and postoperative recovery was uneventful.

Table 1 summarizes the siblings' developmental milestones and clinical signs and symptoms of MPS‐II. To evaluate their development, we used KSPD (K‐test), which is a standard tool in Japan.^14^, ^15^ Developmental quotients (DQ) can be equated with an intelligence Quotient (IQ). A study comparing the development of very low birth weight infants with the Bailey score and the KSPD, both showed similar results in cognitive age equivalents and motor skills^16^ but not language and social skills. Notably, same‐age comparisons show that sibling 2 lacked the developmental delays and other MPS‐II‐related CNS signs and symptoms present in sibling 1. Regarding somatic symptoms, both siblings had histories of inguinal hernia and adenoid vegetation, but only sibling 1 developed hepatomegaly, joint stiffness, and skeletal deformity. As of the time of writing, sibling 2 shows no somatic symptoms of MPS‐II.

**TABLE 1:** Developmental milestones and clinical signs and symptoms of MPS‐II in the two siblings

Age	Sibling 1	Sibling 2
10 m		Walks independently
1 y		Speaks in single words
1 y 3 m		Drinks water from a cup
1 y 8 m	Walks independently	
2 y	Speaks in single words	
2 y 4 m	Delays in language acquisition Mild ventriculomegaly and brain atrophy	
2 y 8 m	Difficulty concentrating Impaired motor and cognitive function Able to go up and down stairs using a handrail	
2 y 11 m		Utters two‐word sentence
3 y 11 m	Utters two‐word sentence	
4 y 3 m		Mild arthrogryposis in finger joints
5 y	Mild ventriculomegaly and brain atrophy (no exacerbation) Unable to go up and down stairs even using a handrail	

**FIGURE 1:**
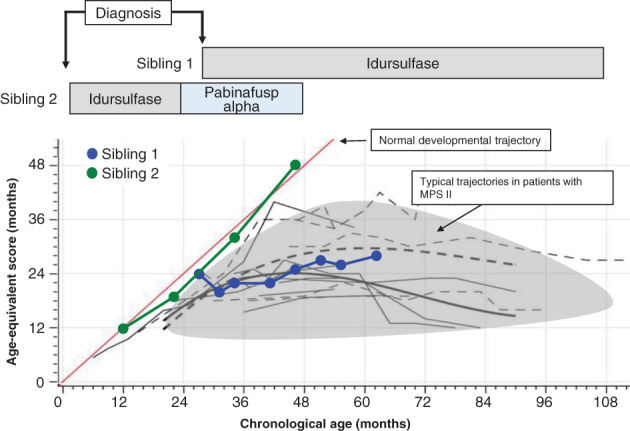
Developmental trajectories and time‐course changes in MPS II symptoms in the two siblings

**FIGURE 2:**
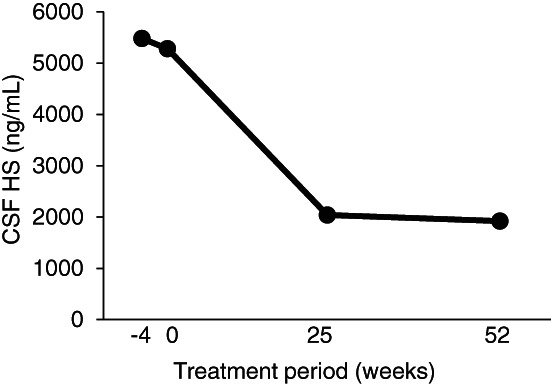
HS concentrations in the CSF in sibling 2 after ERT with pabinafusp alfa

Figure 1 shows the available information on the developmental trajectories and ERT time‐courses in the siblings, which is presented against reported natural history data on the typical course of cognitive development in MPS‐II.^8^ It is noteworthy that sibling 1's trajectory matches that of the typical course, whereas that of sibling 2 is close to that considered to represent normal development, with age‐equivalent scores exceeding those of his chronological age after he became 42 months old (Figure 2).

**FIGURE 3:**
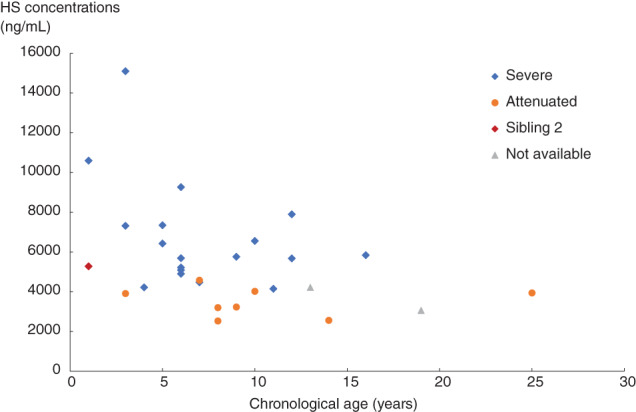
Baseline HS concentrations in the CSF of the 28 patients in the phase II/III clinical trial of pabinafusp alfa

In sibling 2, HS concentrations in the cerebrospinal fluid (CSF) were measured at 4 points in the course of the clinical trial with high‐sensitivity liquid‐chromatography‐tandem mass spectrometry.^17^ The HS concentration rapidly decreased from 5480 ng/mL at the baseline to 2040 ng/mL at week 25 and remained low (1920 ng/mL) at week 52, with an overall reduction of 63.3% over 52 weeks (Supplemental Figure 2). Figure 3 shows a distribution of the baseline HS concentrations in the CSF of the 28 subjects enrolled in the trial, including sibling 2 (shown in red). The trial investigators classified their patients into the severe (neuropathic) and attenuated (non‐neuropathic) subtypes according to their clinical presentations, with most of the neuropathic patients having HS levels higher than 4000 ng/mL.

## 3 DISCUSSION

This report highlights the marked differences in the clinical courses of two Japanese siblings with MPS‐II sharing the same genotype. Phenotypic differences were most notable in the CNS manifestations, in particular the normal neurodevelopmental trajectory of sibling 2 as opposed to that of sibling 1, which matched the course that would typically be expected in such patients.^8^ Also, the critical importance of initiating ERT early is attested by the lack of somatic symptoms in sibling 2 as compared with his older brother, who started receiving ERT at the age of 2 years 4 months. Although long‐term neurocognitive assessment is required to confirm whether and to what extent the apparently normalized developmental trajectory in sibling 2 is sustainable, it seems reasonable to attribute the marked bifurcate neurodevelopmental difference between the siblings observed within 2 years to the early initiation of ERT and to the switching of idursulfase to pabinafusp alfa in sibling 2.

HS concentrations in the CSF are not routinely measured as part of the clinical management of MPS‐II, but the collective distribution of baseline HS concentrations shown in Figure 3 suggests that the concentrations act as a good biomarker of the severity of neurodegeneration, which itself seems to reflect treatment response in terms of CNS manifestations. This was the case in sibling 2, whose normalized neurodevelopmental trajectory was accompanied by a rapid decrease in CSF HS levels. A HS concentration in the CSF of around 4000 ng/mL seems to be the threshold for neurodegeneration: the HS concentrations in the 20 neuropathic patients in the trial were 6626 ± 2700 ng/mL at baseline, and these levels had significantly decreased to 2294 ± 909.9 ng/mL at week 52, by which stage positive neurocognitive changes were evident 1.^12^

ERT has markedly improved the prognosis of patients with MPS‐II, with the risk of death decreased by 54% in patients receiving ERT, as shown by the Hunter Outcome Survey.^7^ This longitudinal evidence points to the critical importance of ERT in overall survival, so early initiation after diagnosis is clearly desirable. However, the same survey also found a fivefold higher risk of death in patients with cognitive impairment than in those without.

Because of the BBB, the CNS manifestations are impervious to conventional ERT, which constitutes a formidable therapeutic challenge. Various efforts have been made to overcome this, including hematopoietic stem cell transplantation,^18^ intrathecal ERT,^19^ and intracerebroventricular ERT.^20^ The notable differences in CNS manifestations that we observed in two siblings with the same genotype demonstrate the salient clinical benefits of ERT with a BBB‐crossing enzyme that can be administered without the practical burdens associated with transplantation or intrathecal/intracerebroventricular administration.

Respiratory failure is the most common cause of death in patients with MPS‐II,^7^ and it is associated with obstructions caused by GAG deposits in the respiratory tract.^21^ Conventional ERT slows but does not stop the progression of airway disease, so even though early initiation of ERT can alleviate airway involvement, persistent respiratory problems still affect long‐term survival.^20^ Because of a high risk of death in patients with neuropathic MPS‐II, some neurological involvement may probably be assumed in the respiratory failure, which itself is primarily based on the airway lesions. Indeed, neurodegeneration is likely to induce delayed reflexes, hypotonia, and generalized discoordination, thereby complicating swallowing function^21^ and increasing the risk of aspiration in patients with preexisting respiratory ailments. ERT with a BBB‐crossing enzyme may, therefore, have the potential to simultaneously address the progression of airway disease and the neurodegeneration that exacerbates it. Further long‐term evidence will clarify how and to what extent early initiation of ERT with a BBB‐crossing enzyme can improve or prevent the CNS manifestations that affect both the quality of life and overall survival of patients with MPS‐II. Therefore, newborn screening and diagnosis of MPS in early phase are meaningful to achieving these aims.

### AUTHORS CONTRIBUTION

Kazuyoshi Tomita, Shungo Okamoto, Toshiyuki Seto, and Takashi Hamazaki all took part in the clinical management of the patients and data collection for this report. Sairei So, Tatsuyoshi Yamamoto, and Kazunori Tanizawa designed and managed the clinical trial in which sibling 2 participated. Kazuyoshi Tomita and Yuji Sato wrote the first manuscript. All authors were involved in the interpretation and critical review of the data, and in drafting and revising the manuscript; all approved the final version proposed by Kazuyoshi Tomita and Yuji Sato.

## Supporting information


**Figure 2** HS concentrations in the CSF in sibling 2 after ERT with pabinafusp alfa
