# Supplementary material for: Divergent developmental trajectories in two siblings with neuropathic mucopolysaccharidosis type II (Hunter syndrome) receiving conventional and novel enzyme replacement therapies: A case report
Source: JIMD Rep. 2021 Jul 27;62(1):9–14. doi: 10.1002/jmd2.12239 (PMC8574176; doi:10.1002/jmd2.12239)
Supplement: Supplementary file 1 — Figure 2 HS concentrations in the CSF in sibling 2 after ERT with pabinafusp alfa [file JMD2-62-9-s001.pptx]

## Slide 1
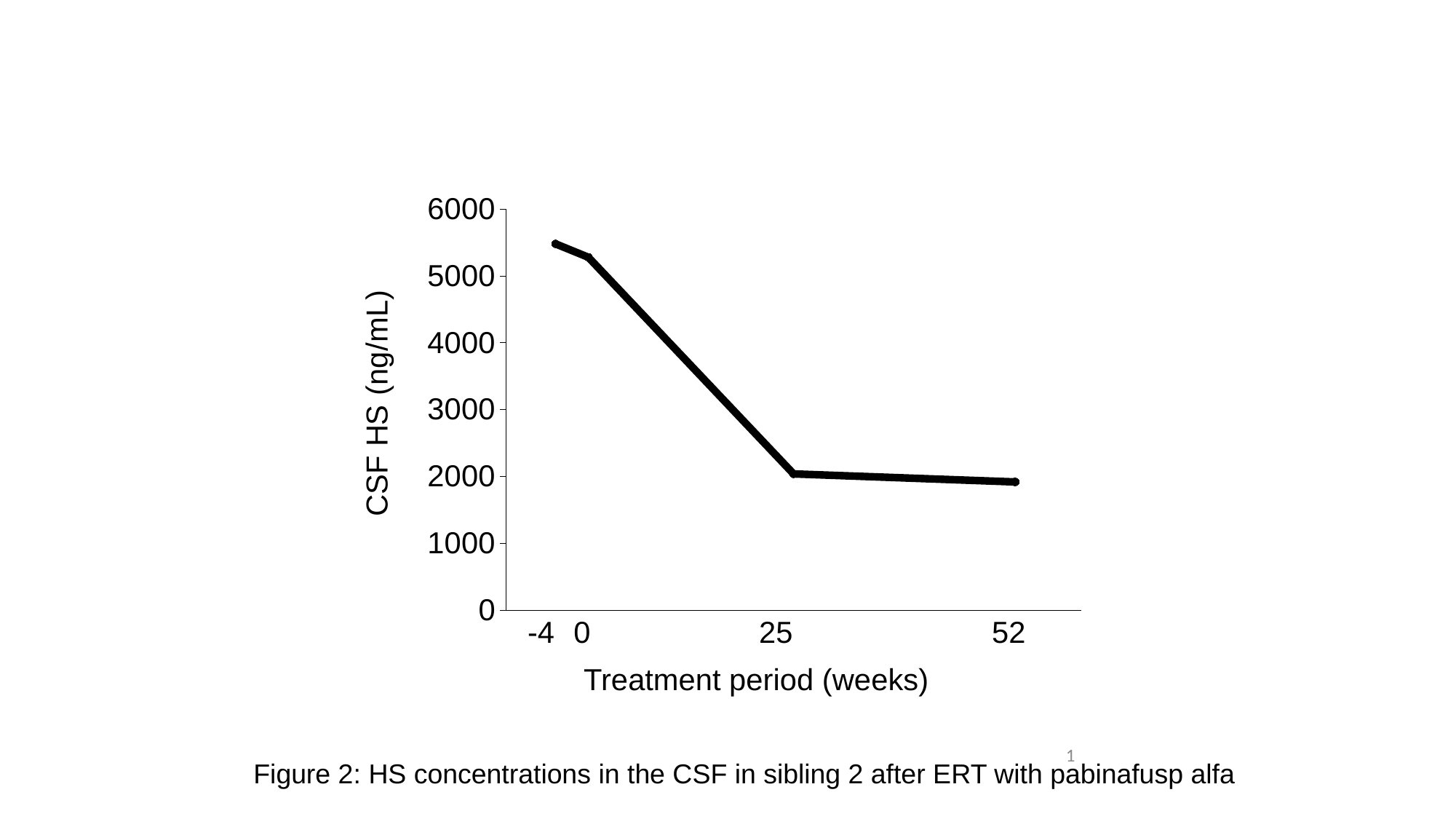

### Chart
| Category | |
|---|---|-4
0
25
52
Treatment period (weeks)
1
Figure 2: HS concentrations in the CSF in sibling 2 after ERT with pabinafusp alfa
